# Serum phoenixin-14 and phoenixin-20 concentrations in polycystic ovary syndrome: Associations with anti-Müllerian hormone and diagnostic utility

**DOI:** 10.17305/bb.2026.14394

**Published:** 2026-06-14

**Authors:** Oznur Dundar Akin, Naile Fevziye Misirlioglu, Seyma Dumur, Mete Hakan Karalok, Yeliz Çeçen Dönmez, Gonul Simsek, Hafize Uzun

**Affiliations:** 1 Department of Obstetrics and Gynecology, Faculty of Medicine, Istanbul Atlas University, Istanbul, Türkiye; 2 Department of Biochemistry, Faculty of Medicine, Istanbul Atlas University, Istanbul, Türkiye; 3 Department of Obstetrics and Gynecology, Kartal Dr. Lütfi Kırdar City Hospital, University of Health Sciences, Istanbul, Türkiye; 4 Department of Physiology, Istanbul Atlas University, Istanbul, Türkiye

**Keywords:** Phoenixin-14, phoenixin-20, polycystic ovary syndrome, PCOS, anti-Müllerian hormone, AMH, mediation analysis, biomarkers

## Abstract

Polycystic ovary syndrome (PCOS) is a complex metabolic disorder characterized by altered peptide signaling that may contribute to reproductive dysfunction, insulin resistance, and metabolic dysregulation. This case-control study investigated serum phoenixin (PNX) isoforms, phoenixin-14 (PNX-14) and phoenixin-20 (PNX-20), in women with PCOS to evaluate their associations with biochemical and hormonal markers, including anti-Müllerian hormone (AMH), and assess their diagnostic utility. Serum PNX-14 and PNX-20 levels were measured in 90 women with PCOS and 60 age-matched healthy controls. Demographic, metabolic, and hormonal parameters were compared between the groups. Statistical analyses included receiver operating characteristic (ROC) analysis, logistic regression, correlation analysis, principal component analysis (PCA)-based clustering, mediation analysis, and exploratory Random Forest analysis. Results indicated that PNX-14 and PNX-20 levels were significantly higher in women with PCOS compared to controls (*P <* 0.001). Both isoforms correlated positively with AMH, insulin, homeostatic model assessment for insulin resistance (HOMA-IR), and luteinizing hormone (LH), while demonstrating negative correlations with sex hormone-binding globulin (SHBG) and high-density lipoprotein (HDL) cholesterol. ROC analysis showed moderate discriminatory performance, with area under the curve (AUC) values of 0.834 for PNX-14 and 0.819 for PNX-20. Mediation analysis suggested an associative pattern among PNX isoforms, AMH, and PCOS status. PCA-based clustering identified two distinct phenotypic profiles. Random Forest analysis ranked AMH, SHBG, fasting insulin, and body mass index (BMI) as the most influential variables, with PNX isoforms demonstrating lower importance scores. In conclusion, circulating PNX-14 and PNX-20 were elevated in PCOS and associated with key reproductive and metabolic parameters, suggesting a potential complementary role within multi-marker assessment frameworks. However, given the cross-sectional design and exploratory nature of the analyses, these findings should be considered hypothesis-generating rather than causal. Future longitudinal, mechanistic, and externally validated multicenter studies are essential to further elucidate these relationships.

## Introduction

Polycystic ovary syndrome (PCOS) is a complex endocrine and metabolic disorder affecting women of reproductive age. It is increasingly recognized as a systemic condition characterized by insulin resistance and metabolic dysregulation. Insulin resistance is a central component of PCOS pathophysiology, contributing to compensatory hyperinsulinemia, hyperandrogenism, and reproductive dysfunction. The clinical spectrum of PCOS is broad, encompassing metabolic syndrome, type 2 diabetes mellitus (T2DM), cardiovascular disease (CVD), and metabolic dysfunction–associated steatotic liver disease (MAFLD), among other comorbidities. The prevalence and phenotypic presentation of PCOS may vary by ethnic background and clinical phenotype. Despite its chronic and heterogeneous nature, modest lifestyle interventions, including an approximate 5% reduction in body weight, have been associated with significant improvements in metabolic and reproductive outcomes [1–5].

The pathogenesis of PCOS is multifactorial, involving complex interactions among metabolic, hormonal, and neuroendocrine factors. Dysregulation of the hypothalamic–pituitary–ovarian (HPO) axis, particularly increased luteinizing hormone (LH) secretion, is a key contributor to ovarian dysfunction in PCOS. Additionally, elevated anti-Müllerian hormone (AMH) levels have been implicated in impaired follicular maturation and ovulatory dysfunction. A characteristic feature of PCOS is the bidirectional interaction between hyperinsulinemia and androgen excess, which may contribute to a self-perpetuating cycle of metabolic and reproductive disturbances. These interconnected mechanisms further support the concept of PCOS as a systemic neuroendocrine–metabolic disorder rather than a condition limited solely to ovarian dysfunction [6, 7].

Phoenixin (PNX), an endogenous peptide, was first identified in the rat brain by Yosten et al. in 2013 [8]. Subsequent studies have demonstrated its expression across multiple species, including humans. PNX primarily exists in two biologically active isoforms, PNX-14 and PNX-20, composed of 14 and 20 amino acids, respectively. Experimental evidence suggests that PNX may regulate reproductive function by modulating gonadotropin-releasing hormone (GnRH) and luteinizing hormone secretion. Furthermore, PNX has been implicated in various physiological processes, including nociception, anxiety-related behavior, cognitive function, and metabolic regulation. Recent research also suggests potential anti-inflammatory and metabolic regulatory properties of PNX, though its precise biological role in human metabolic disorders remains incompletely understood [9–13].

Despite growing interest in neuroendocrine peptides in metabolic disorders, clinical data on circulating PNX isoforms in women with PCOS are limited. Specifically, the relationships between PNX-14 and PNX-20 and key reproductive and metabolic markers, such as AMH, gonadotropins, insulin resistance indices, and lipid parameters, have not been comprehensively evaluated.

Therefore, the present study was designed to investigate serum PNX-14 and PNX-20 levels in women with PCOS compared to healthy controls and to evaluate their relationships with insulin resistance indices and hormonal parameters. We hypothesized that altered PNX isoform levels may be associated with the metabolic and reproductive features of PCOS. The study further aimed to explore potential associations between phoenixin isoforms, AMH-related reproductive characteristics, and metabolic parameters within a well-characterized PCOS cohort.

## Materials and methods

### Study design and ethical approval

The study adhered to the ethical principles of the Declaration of Helsinki and received approval from the Istanbul Atlas University Health Sciences Scientific Research Ethics Committee (approval date: October 30, 2025; reference no: E-22686390-050.99-80742). All participants were thoroughly informed about the study objectives and procedures before participation, and electronic informed consent was obtained.

### Study population

The study population comprised women diagnosed with PCOS and age-matched healthy control subjects, all of Turkish origin. Participants were recruited from a single tertiary care center, Istanbul Atlas University Hospital (Istanbul, Turkey), between November 2025 and January 31, 2026. Age matching within ±2 years was applied during recruitment to ensure comparable age distributions between groups. However, strict one-to-one individual matching was not performed; thus, the matching strategy is considered frequency-based rather than pairwise. Although an initial 1:1 allocation strategy was planned, final sample sizes differed due to recruitment constraints and stricter inclusion/exclusion criteria applied to the control group. Importantly, the dataset was not analyzed as individually paired, and no paired statistical methods were applied. This approach ensured well-defined and well-characterized control groups.

PCOS was diagnosed according to the revised Rotterdam criteria, requiring the presence of at least two of the following findings after excluding related disorders: (i) oligo/anovulation, (ii) clinical and/or biochemical hyperandrogenism, and/or (iii) polycystic ovarian morphology on ultrasonography [14]. Oligo/anovulation was defined as menstrual cycles longer than 35 days or fewer than eight menstrual cycles per year. Clinical hyperandrogenism was assessed by the presence of hirsutism, acne, or androgenic alopecia, while biochemical hyperandrogenism was defined by elevated serum androgen levels above the laboratory-specific reference range. Polycystic ovarian morphology was defined by ultrasonographic detection of ≥12 follicles measuring 2–9 mm in diameter and/or an ovarian volume >10 mL in at least one ovary.

### Inclusion and exclusion criteria

#### Inclusion criteria

Participants were eligible if they met all the following criteria:

(i) Female sex, aged 18–40 years; (ii) For the PCOS group, a confirmed diagnosis of PCOS according to the Rotterdam criteria; (iii) For the control group, regular menstrual cycles, absence of clinical or biochemical hyperandrogenism, and normal ovarian morphology; (iv) Willingness to participate and provision of written informed consent.

#### Exclusion criteria

Participants were excluded if they had any of the following conditions:

(i) Pregnancy or lactation; (ii) Known thyroid disorders, hyperprolactinemia, congenital adrenal hyperplasia, Cushing’s syndrome, or androgen-secreting tumors; (iii) Type 1 or type 2 diabetes mellitus, chronic liver or kidney disease, or established cardiovascular disease; (iv) Use of hormonal contraceptives, insulin-sensitizing agents, glucocorticoids, anti-androgens, or lipid-lowering medications within the preceding three months; (v) Active inflammatory or autoimmune disease, malignancy, or acute infection.

### Data collection and laboratory measurements

Potential sources of bias were minimized through consecutive recruitment of eligible participants using predefined inclusion and exclusion criteria. Age matching (±2 years) was applied, and age was additionally adjusted for in multivariable analyses to reduce residual confounding. Standardized protocols were used for clinical assessment and laboratory measurements to limit measurement bias.

Demographic characteristics, anthropometric data, and clinical history were recorded for all participants at enrollment. Fasting blood samples were collected after an overnight fast to evaluate glucose, insulin, lipid profile, reproductive hormones, and serum PNX-14 and PNX-20 levels. Insulin resistance was calculated using the homeostatic model assessment for insulin resistance (HOMA-IR), derived from fasting glucose and insulin concentrations.

### Sample collection and biochemical parameters

Blood samples were collected in the morning after an overnight fast. In healthy controls with regular menstrual cycles, hormonal measurements were obtained during the early follicular phase (days 2–5 of the menstrual cycle). In women with PCOS experiencing irregular menstrual cycles or amenorrhea, blood sampling was performed either during spontaneous bleeding episodes or at a randomly selected morning visit, consistent with common clinical practice in PCOS research.

Venous blood samples were obtained in the morning after an overnight fasting period of at least 8 h. Serum was separated by centrifugation at 3000 rpm for 10 min, aliquoted into sterile tubes, and stored at −80 ^∘^C until analysis. Repeated freeze–thaw cycles were avoided, and all samples underwent a single thaw before measurement. ELISA analyses were performed in duplicate, and samples from PCOS and control groups were analyzed using the same assay batches whenever possible to minimize inter-assay variability. Serum samples were diluted according to the manufacturer’s recommendations when required. Laboratory personnel performing the measurements were blinded to participant group allocation.

Routine biochemical parameters, including fasting blood glucose and lipid profile (high-density lipoprotein cholesterol, low-density lipoprotein cholesterol, and triglycerides), were measured using an automated clinical chemistry analyzer (Cobas 8000, Roche Diagnostics, Mannheim, Germany). Serum insulin levels were determined by electrochemiluminescence immunoassay (ECLIA) using the same platform.

Hormonal parameters, including LH, follicle-stimulating hormone (FSH), estradiol, prolactin, TSH, dehydroepiandrosterone sulfate (DHEA-S), 17-hydroxyprogesterone (17-OHP), sex hormone–binding globulin (SHBG), and anti-Müllerian hormone (AMH), were measured using automated immunoassay systems (Cobas e601 analyzer, Roche Diagnostics, Mannheim, Germany) in accordance with the manufacturer’s protocols.

### Measurement of serum phoenixin-14 and phoenixin-20

Serum phoenixin-14 (PNX-14) and phoenixin-20 (PNX-20) concentrations were quantified using commercially available sandwich ELISA kits (FineTest, Wuhan Fine Biotech Co., Ltd., Wuhan, Hubei, China; Catalog No. EH4521 and EH4352; RRID: AB-3665841 and AB-3665827, respectively). The analytical sensitivities were 0.938 pg/mL for PNX-14 and 9.375 pg/mL for PNX-20. The assay ranges were 1.563–100 pg/mL for PNX-14 and 15.625–1000 pg/mL for PNX-20. Manufacturer-reported intra-assay and inter-assay coefficients of variation were <8% and <10%, respectively.

Prior to analysis, serum samples were diluted 1:2 with the provided sample dilution buffer, adhering to the manufacturer’s recommendations. Samples were aliquoted, stored at −80^∘^C, and analyzed within three months of collection. All measurements were performed in duplicate, following the manufacturer’s instructions. Absorbance was recorded at 450 nm using an Epoch™ Microplate Reader (BioTek Instruments, Winooski, VT, USA). Concentrations were derived from standard curves and adjusted for the dilution factor. The mean of duplicate measurements was used for all statistical analyses. Standardized laboratory procedures were rigorously applied to minimize analytical variability and enhance reproducibility.

### Statistical analysis

All statistical analyses were conducted using IBM SPSS Statistics for Windows, Version 25.0 (IBM Corp., Armonk, NY, USA) and Python (version 3.9) for advanced modeling and visualization. Data distribution was evaluated using the Kolmogorov–Smirnov and Shapiro–Wilk tests, complemented by visual inspection of histograms. Continuous variables were presented as mean ± standard deviation (SD) for normally distributed data or as median (interquartile range, IQR) for non-normally distributed data. Normally distributed continuous variables were compared using Student’s *t*-test, while non-normally distributed variables were compared using the Mann–Whitney *U* test. Categorical variables were expressed as frequencies and percentages, and comparisons were made using the chi-square test or Fisher’s exact test, as appropriate.

Although age matching (±2 years) was implemented during the study design, age was additionally included as a covariate in all multivariable analyses to mitigate potential residual confounding. Multivariable logistic regression analyses were performed to assess the associations between phoenixin isoforms and PCOS status, with results reported as odds ratios (ORs) and 95% confidence intervals (CIs). A predefined core adjustment set, comprising age, BMI, and HOMA-IR, was selected *a priori* due to their established associations with both PCOS and metabolic dysfunction. Additional candidate variables demonstrating potential associations in univariable analyses were considered during model construction.

Receiver operating characteristic (ROC) curve analyses were conducted to evaluate the discriminative performance of PNX isoforms for PCOS. Optimal cutoff values were determined using the Youden index. Sensitivity, specificity, area under the curve (AUC), and corresponding 95% confidence intervals (CIs) were calculated.

Correlation analyses were primarily performed within the PCOS group to assess associations between phoenixin isoforms and clinical/biochemical parameters. Pearson or Spearman correlation analyses were applied based on data distribution characteristics.

Principal component analysis (PCA) and k-means clustering were utilized to identify exploratory data-driven clusters within the study population.

Mediation analysis, employing the Baron and Kenny approach, explored the statistical association involving AMH, PNX isoforms, and PCOS status. Given the cross-sectional case–control design, these analyses were interpreted as exploratory and associative rather than causal.

Random Forest analysis was performed to identify variables most strongly associated with PCOS and to provide exploratory feature ranking. As the primary objective was not predictive model development or validation, train/test splitting, cross-validation, and external validation procedures were not undertaken. Consequently, the resulting variable-importance rankings should be interpreted as exploratory and hypothesis-generating rather than as evidence of predictive performance.

A *P*-value < 0.05 was considered statistically significant.

## Results

A total of 238 women were assessed for eligibility. After excluding 88 women who did not meet the inclusion criteria or had incomplete data, 150 participants (90 women with PCOS and 60 healthy controls) were included in the final analysis (Figure 1).

**Figure 1.**
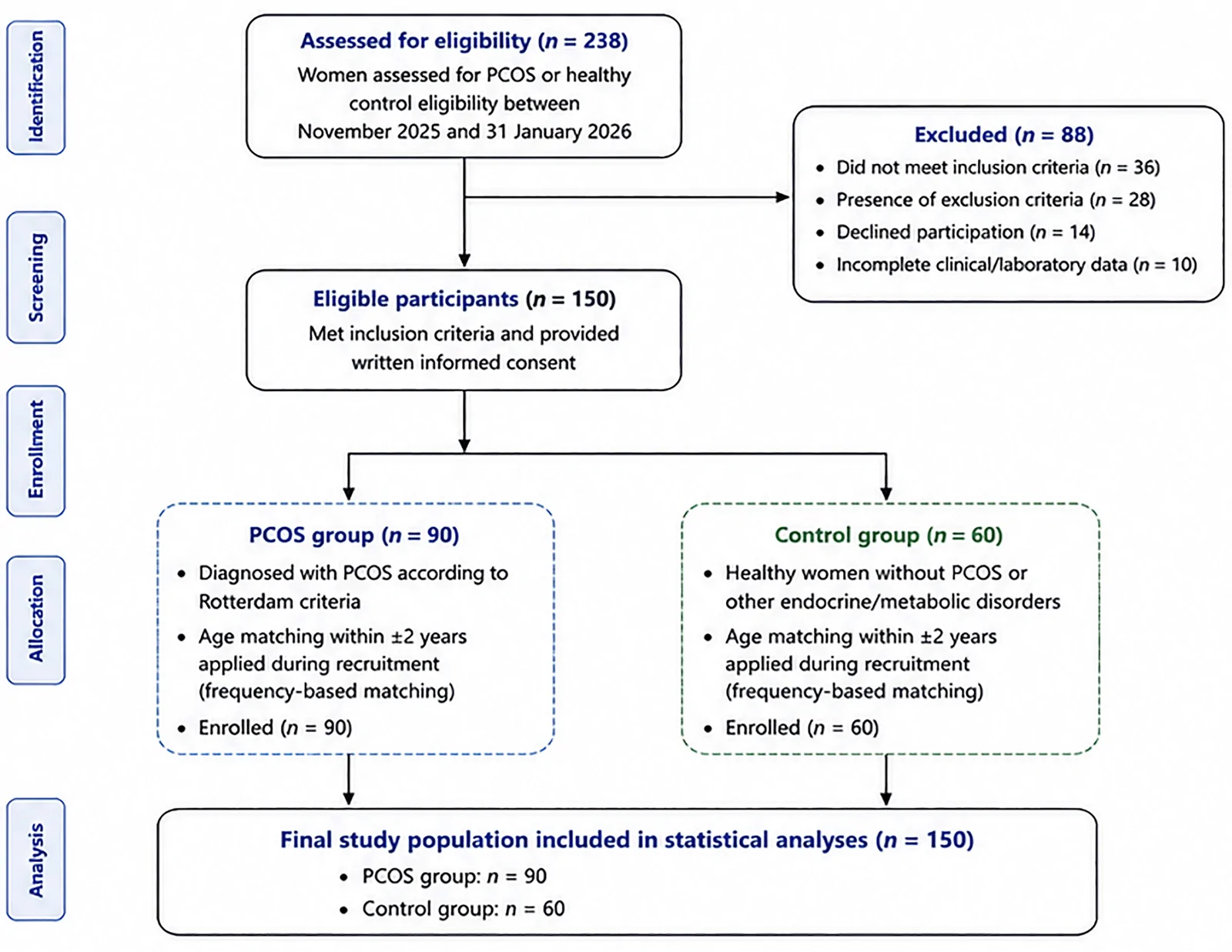
**Flow diagram of participant selection, screening, exclusion, and inclusion in the final analysis.** **Abbreviation:** PCOS, polycystic ovary syndrome.

Baseline demographic, metabolic, and hormonal characteristics of the study population are presented in Table 1. Detailed phenotype-based subgroup analyses according to the Rotterdam classification were not available for all participants and were therefore not included in the present study. The PCOS group exhibited significantly higher body mass index (BMI), fasting insulin levels, HOMA-IR, AMH, LH, DHEA-S, 17-OHP, triglycerides (TG), LDL cholesterol, and serum PNX-14 and PNX-20 concentrations compared with the control group (all *P <* 0.001). Conversely, FSH, SHBG, and HDL cholesterol levels were significantly lower in women with PCOS. Although TSH levels differed statistically between groups, their absolute values remained within the normal reference range. No statistically significant differences were observed between groups for age, estradiol (E2), or prolactin (PRL) levels (Table 1).

**Table 1:** Baseline demographic and biochemical characteristics

**Variable**	**PCOS (*n* ═ 90)** **(mean ± SD)**	**Controls (*n* ═ 60)** **(mean ± SD)**	***P* value**
Age (years)	26.80 ± 5.34	27.92 ± 4.87	0.187
BMI (kg/m^2^)	27.96 ± 3.92	22.06 ± 2.92	<0.001
Fasting blood glucose (mg/dL)	96.61 ± 11.18	93.52 ± 11.22	0.099
Fasting insulin (µIU/mL)	18.56 ± 6.42	9.22 ± 3.09	<0.001
HOMA-IR (unitless)	4.42 ± 1.60	2.13 ± 0.77	<0.001
Anti-Müllerian hormone (ng/mL)	6.72 ± 1.99	2.83 ± 0.84	<0.001
Follicle-stimulating hormone (IU/L)	4.84 ± 1.63	6.10 ± 1.19	<0.001
Luteinizing hormone (IU/L)	8.77 ± 2.65	4.83 ± 1.55	<0.001
Estradiol (pg/mL)	46.67 ± 14.92	46.58 ± 16.53	0.973
Sex hormone–binding globulin (nmol/L)	29.89 ± 8.19	53.65 ± 12.68	<0.001
Dehydroepiandrosterone sulfate (µg/dL)	313.13 ± 107.75	172.07 ± 53.92	<0.001
17-Hydroxyprogesterone (ng/mL)	1.80 ± 0.57	1.12 ± 0.42	<0.001
Thyroid-stimulating hormone (µIU/mL)	1.94 ± 0.80	2.23 ± 0.89	0.042
Prolactin (ng/mL)	13.20 ± 5.30	14.12 ± 5.21	0.297
Triglycerides (mg/dL)	161.26 ± 37.85	107.09 ± 28.38	<0.001
High-density lipoprotein cholesterol (mg/dL)	43.37 ± 7.26	57.76 ± 9.72	<0.001
Low-density lipoprotein cholesterol (mg/dL)	140.97 ± 33.12	105.47 ± 28.34	<0.001
Phoenixin-14 (pg/mL)	221.95 ± 56.49	150.50 ± 43.67	<0.001
Phoenixin-20 (pg/mL)	174.07 ± 53.89	115.91 ± 34.73	<0.001

Values are presented as mean ± standard deviation. Normality was assessed using the Shapiro–Wilk test. Variables with a normal distribution were compared using the independent samples *t*-test, whereas non-normally distributed variables were compared using the Mann–Whitney *U* test. Effect sizes were calculated using Cohen’s d and interpreted as small (0.2), moderate (0.5), and large (0.8). All tests were two-tailed, and a *P*-value < 0.05 was considered statistically significant. **Abbreviations:** BMI, body mass index; HOMA-IR, homeostatic model assessment for insulin resistance; PCOS, polycystic ovary syndrome; SD, standard deviation.

Serum concentrations of PNX-14 and PNX-20 were markedly elevated in the PCOS group compared with controls, as illustrated in Figure 2. Mean PNX-14 levels were 221.95 ± 56.49 pg/mL in the PCOS group vs 150.50 ± 43.67 pg/mL in controls, while PNX-20 levels were 174.07 ± 53.89 pg/mL and 115.91 ± 34.73 pg/mL, respectively (both *P <* 0.001).

**Figure 2.**
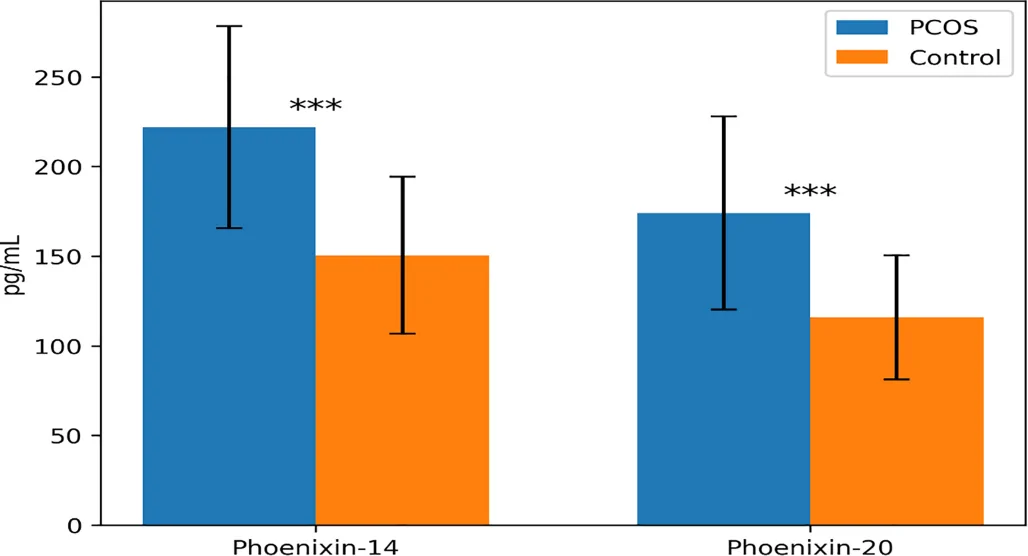
**Serum phoenixin-14 and phoenixin-20 concentrations in women with PCOS and healthy controls.** Women with PCOS (*n* ═ 90) had significantly higher serum phoenixin-14 levels than healthy controls (221.95 ± 56.49 vs. 150.50 ± 43.67 pg/mL, respectively; *P <* 0.001). Similarly, serum phoenixin-20 levels were significantly elevated in the PCOS group compared with controls (174.07 ± 53.89 vs. 115.91 ± 34.73 pg/mL, respectively; *P <* 0.001). Data are presented as mean ± SD, and error bars indicate SD ***P < 0.001.. **Abbreviations:** PCOS, polycystic ovary syndrome; SD, standard deviation.

Cluster analysis identified two exploratory data-driven clusters. Cluster 0 comprised 62 participants (60 PCOS, 2 controls), whereas Cluster 1 included 88 participants (30 PCOS, 58 controls). The distribution of PCOS and control participants differed between clusters. Cluster-based profiling revealed distinct biochemical patterns, with higher PNX, BMI, and AMH levels observed in the cluster with a greater proportion of women with PCOS. Detailed cluster characteristics are presented in Table 2, and the corresponding PCA-based distribution is illustrated in Figure 3. These exploratory clusters should not be interpreted as established PCOS phenotypes or Rotterdam subtypes. Given that clustering variables were also used in cluster interpretation, these findings should be interpreted cautiously and considered exploratory.

**Table 2:** Cluster-based comparison of key biochemical parameters

**Variable**	**Cluster 0**	**Cluster 1**	***P*-value**	**Cohen’s *d***
Phoenixin–14 (pg/mL)	250.45 ± 39.88	153.15 ± 39.95	1.011 × 10^−30^	2.44
Phoenixin–20 (pg/mL)	186.81 ± 49.68	125.44 ± 43.40	2.794 × 10^−13^	1.33
BMI (kg/m^2^)	27.88 ± 3.99	24.00 ± 4.30	9.727 × 10^−8^	0.93
AMH (ng/mL)	6.54 ± 2.12	4.19 ± 2.31	2.778 × 10^−9^	1.05

Values are presented as mean ± standard deviation. Comparisons between clusters were performed using the independent samples *t*-test. Effect sizes were calculated using Cohen’s d and interpreted as small (0.2), moderate (0.5), and large (0.8). **Abbreviations:** AMH, anti-Müllerian hormone; BMI, body mass index.

**Figure 3.**
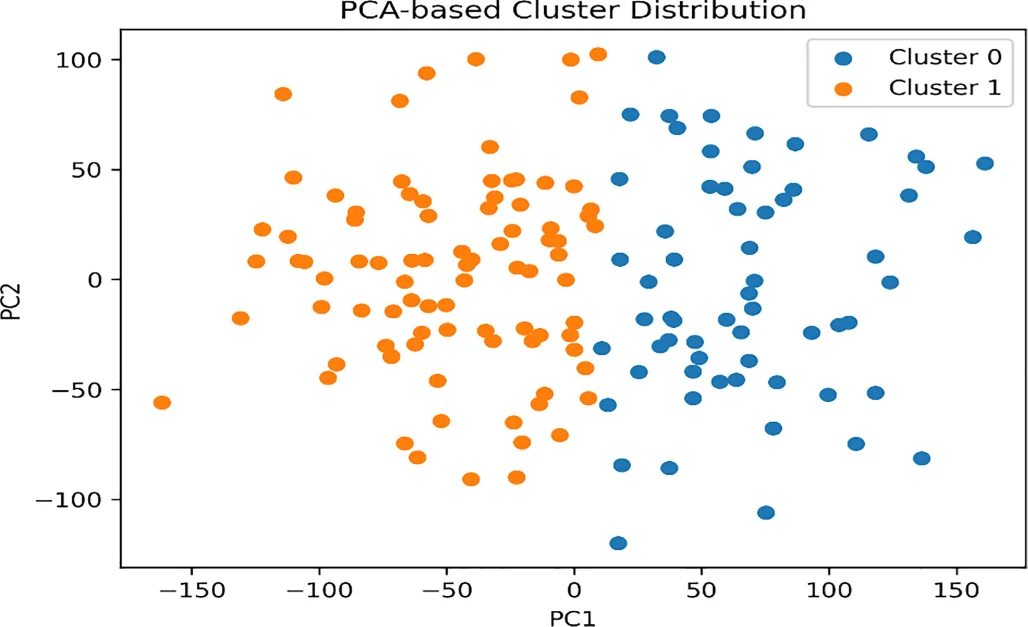
**PCA–based visualization of exploratory cluster distribution in the overall study population.** Each point represents one participant from the combined study population of women with PCOS and healthy controls (*n* ═ 150). PCA was used to visualize the distribution of participants according to k-means cluster assignment based on serum PNX-14, PNX-20, BMI, and AMH levels. Cluster 0 included 62 participants, predominantly women with PCOS (60 PCOS and 2 controls), whereas Cluster 1 included 88 participants, predominantly healthy controls (30 PCOS and 58 controls). PC1 and PC2 represent the first and second principal components, respectively. These clusters should be interpreted as exploratory data-driven patterns and not as established PCOS phenotypes or Rotterdam subtypes. **Abbreviations:** AMH, anti-Müllerian hormone; BMI, body mass index; PCA; Principal component analysis; PCOS, polycystic ovary syndrome; PNX-14, phoenixin-14; PNX-20, phoenixin-20.

**Figure 4.**
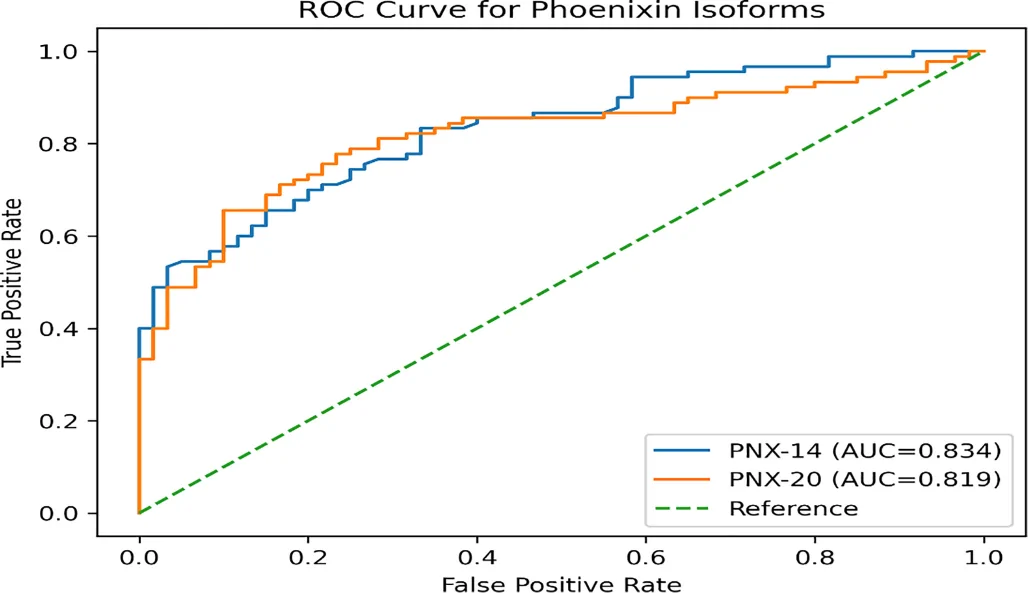
**ROC curves for serum PNX isoforms in discriminating women with polycystic ovary syndrome from healthy controls.** ROC curves show the diagnostic performance of serum PNX-14 and PNX-20 for distinguishing women with PCOS (*n* ═ 90) from age-matched healthy controls (*n* ═ 60). PNX-14 demonstrated an AUC of 0.834, with 66% sensitivity and 85% specificity at the optimal cut-off value of 202.8 pg/mL. PNX-20 demonstrated an AUC of 0.819, with 66% sensitivity and 90% specificity at the optimal cut-off value of 157.4 pg/mL. Optimal cut-off values were determined using the Youden index. The dashed diagonal line represents the no-discrimination reference line. These findings indicate moderate discriminatory performance and should be interpreted as exploratory because cut-off values were derived and evaluated within the same study population. **Abbreviations:** AUC, area under the curve; PNX, phoenixin; PNX-14, phoenixin-14; PNX-20, phoenixin-20; ROC, receiver operating characteristic.

ROC curve analyses demonstrated that both PNX-14 and PNX-20 exhibited significant discriminative ability for PCOS (Figure 4). As the cutoff values were both derived and evaluated within the same study population, these findings should be considered exploratory. Optimal cutoff values were determined using the Youden index. PNX-14 demonstrated an AUC of 0.834 (95% CI: 0.769–0.894) with 66% sensitivity and 85% specificity at a cutoff value of 202.8 pg/mL. PNX-20 demonstrated an AUC of 0.819 (95% CI: 0.748–0.885) with 66% sensitivity and 90% specificity at a cutoff value of 157.4 pg/mL (Table 3).

**Table 3:** ROC curve analysis of phoenixin isoforms for discrimination of PCOS

**Marker**	**AUC (95% CI)**	**Cut-off (pg/mL)**	**Sensitivity (%)**	**Specificity (%)**
Phoenixin-14	0.834 (0.769–0.894)	202.8	65.6 (54.8–75.3)	85.0 (73.4–92.9)
Phoenixin-20	0.819 (0.748–0.885)	157.4	65.6 (54.8–75.3)	90.0 (79.5–96.2)

Optimal cut-off values were determined using the Youden index. Sensitivity and specificity confidence intervals were calculated using the exact (Clopper-Pearson) binomial method. The reported cutoff values were derived and evaluated within the same study population; therefore, they should be considered exploratory. **Abbreviations:** AUC, area under the curve; CI, confidence interval; PCOS, polycystic ovary syndrome; ROC, receiver operating characteristic.

Multivariable logistic regression analysis indicated that both PNX-14 and PNX-20 remained significantly associated with PCOS after adjustment for age, BMI, and HOMA-IR (Table 4). Specifically, each 1 pg/mL increase in PNX-14 was associated with a 3.0% increase in the odds of PCOS (adjusted OR: 1.030, 95% CI: 1.001–1.061, *P* ═ 0.044), whereas each 1 pg/mL increase in PNX-20 was associated with a 4.7% increase in the odds of PCOS (adjusted OR: 1.047, 95% CI: 1.016–1.080, *P* ═ 0.003). BMI and HOMA-IR also remained significant predictors of PCOS in the adjusted model.

**Table 4:** Adjusted logistic regression analysis evaluating factors associated with PCOS

**Variable**	**OR**	**Standard error**	*P* **-value**	**95% CI (lower)**	**95% CI (upper)**
Age (years)	0.869	0.110	0.200	0.701	1.077
BMI (kg/m^2^)	2.744	0.318	0.002	1.471	5.119
HOMA-IR	20.142	0.905	<0.001	3.416	118.753
Phoenixin-14 (pg/mL)	1.030	0.015	0.044	1.001	1.061
Phoenixin-20 (pg/mL)	1.047	0.016	0.003	1.016	1.080

Odds ratios are presented per unit increase in each variable. The multivariable logistic regression model included age, BMI, HOMA-IR, Phoenixin-14, and Phoenixin-20 as covariates. After adjustment, both Phoenixin-14 and Phoenixin-20 remained significantly associated with PCOS. **Abbreviations:** BMI, body mass index; CI, confidence interval; HOMA-IR, homeostasis model assessment of insulin resistance; OR, odds ratio; PCOS, polycystic ovary syndrome.

Correlation analysis was performed in the overall study population (PCOS and controls combined; *n* ═ 150). The correlations between serum PNX isoforms and metabolic and hormonal parameters are summarized in Table 5. The strongest correlation was observed between PNX-14 and AMH (*r* ═ 0.43, *P <* 0.001) (Figure 5).

**Figure 5.**
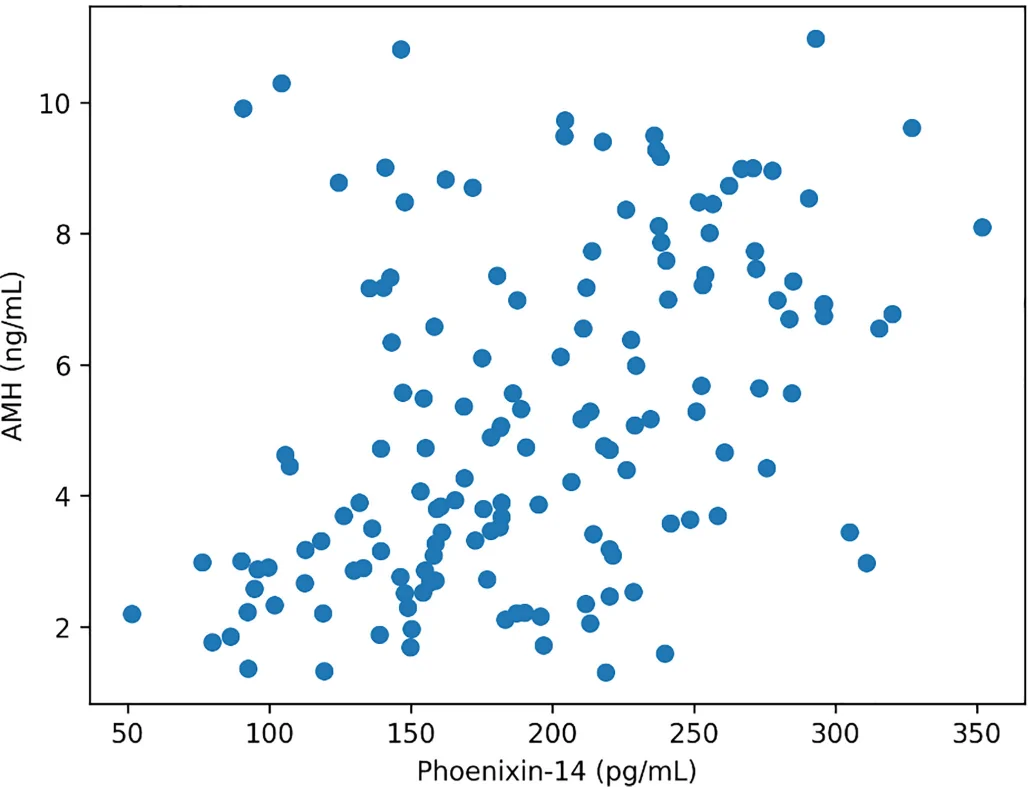
**Correlation between serum PNX-14 and AMH levels in the overall study population (PCOS and controls combined; *n* ═ 150).** The scatter plot shows the relationship between serum PNX-14 and AMH concentrations in the combined study population, including women with PCOS and healthy controls (*n* ═ 150). Each point represents one participant. Pearson correlation analysis demonstrated a moderate positive correlation between PNX-14 and AMH levels (*r* ═ 0.43, *P <* 0.001), suggesting that higher circulating PNX-14 concentrations were associated with higher AMH levels. This association should be interpreted as exploratory and does not imply causality. **Abbreviations:** AMH, anti-Müllerian hormone; PCOS, polycystic ovary syndrome; PNX-14, phoenixin-14.

Mediation analysis, employing the three-step Baron and Kenny approach, investigated the statistical association involving PNX isoforms, AMH, and PCOS status. PNX-14 levels were significantly associated with PCOS status (β ═ 0.0044, *P <* 0.001) and showed a significant positive association with AMH levels (β ═ 0.017, *P <* 0.001). When both PNX-14 and AMH were included in the model, both variables remained significantly associated with PCOS status (PNX-14: β ═ 0.002, *P <* 0.001; AMH: β ═ 0.125, *P <* 0.001). These findings suggest an associative pattern involving PNX-14, AMH, and PCOS status. Given the cross-sectional study design, these results should be interpreted as exploratory and associative rather than as evidence of causal mediation.

Similarly, PNX-20 levels were positively associated with PCOS (β ═ 0.005, *P <* 0.001) and with AMH levels (β ═ 0.018, *P <* 0.001). In the final model, both PNX-20 (β ═ 0.002, *P <* 0.001) and AMH (β ═ 0.129, *P <* 0.001) remained independently associated with PCOS, further supporting a partial mediating role of AMH in the PNX–PCOS relationship.

Random Forest analysis was performed to identify the most important variables associated with PCOS (Table 6 and Figure 6). This analysis was conducted for exploratory feature-ranking purposes rather than as a validated predictive model. As internal validation procedures, including train/test splitting and cross-validation, were not performed, the reported feature-importance rankings should be interpreted cautiously. AMH, SHBG, fasting insulin, and BMI emerged as the most influential variables, whereas PNX-14 and PNX-20 demonstrated lower relative importance scores (0.013 and 0.011, respectively), indicating a comparatively modest contribution relative to established metabolic and hormonal markers.

**Table 5:** Correlation analysis of PNX-14 and PNX-20 with metabolic and hormonal parameters in the overall study population (*n* ═ 150)

**Variable**	**PNX-14 (*r*)**	***P*-value**	**PNX-20 (r)**	***P*-value**
BMI (kg/m^2^)	0.39	<0.001	0.34	<0.001
Fasting insulin (µIU/mL)	0.36	<0.001	0.27	<0.001
HOMA-IR	0.40	<0.001	0.24	0.003
AMH (ng/mL)	0.43	<0.001	0.39	<0.001
LH (IU/L)	0.35	<0.001	0.35	<0.001
DHEA-S (µg/dL)	0.35	<0.001	0.35	<0.001
17-OHP (ng/mL)	0.38	<0.001	0.26	<0.001
Triglycerides (mg/dL)	0.38	<0.001	0.37	<0.001
SHBG (nmol/L)	−0.38	<0.001	−0.35	<0.001
HDL-C (mg/dL)	−0.33	<0.001	−0.31	<0.001

Pearson correlation analyses were performed on the overall study population, which included both individuals with PCOS and controls (*n* ═ 150). Correlation coefficients were subsequently calculated. **Abbreviations:** 17-OHP, 17-hydroxyprogesterone; AMH, anti-Müllerian hormone; BMI, body mass index; DHEA-S, dehydroepiandrosterone sulfate; HDL-C, high-density lipoprotein cholesterol; HOMA-IR, homeostatic model assessment for insulin resistance; LH, luteinizing hormone; PCOS, polycystic ovary syndrome; PNX-14, phoenixin-14; PNX-20, phoenixin-20; r, Pearson correlation coefficient; SHBG, sex hormone–binding globulin.

**Table 6:** Exploratory Random Forest feature-importance ranking of variables associated with PCOS

**Variable**	**Importance score**
Anti-Müllerian hormone (ng/mL)	0.220
Sex hormone-binding globulin (nmol/L)	0.139
Fasting insulin (µIU/mL)	0.094
Body weight (kg)	0.080
High-density lipoprotein cholesterol (mg/dL)	0.077
Dehydroepiandrosterone sulfate (µg/dL)	0.076
HOMA-IR	0.069
Luteinizing hormone (IU/L)	0.056
Body mass index (kg/m^2^)	0.054
17-Hydroxyprogesterone (ng/mL)	0.047
Low-density lipoprotein cholesterol (mg/dL)	0.027
Phoenixin-14 (pg/mL)	0.013
Triglycerides (mg/dL)	0.012
Phoenixin-20 (pg/mL)	0.011
Follicle-stimulating hormone (IU/L)	0.007
Thyroid-stimulating hormone (µIU/mL)	0.004
Estradiol (pg/mL)	0.003
Age (years)	0.003
Prolactin (ng/mL)	0.003
Height (m)	0.003
Fasting blood glucose (mg/dL)	0.002

**Abbreviations:** HOMA-IR, homeostatic model assessment for insulin resistance; PCOS, polycystic ovary syndrome.

**Figure 6.**
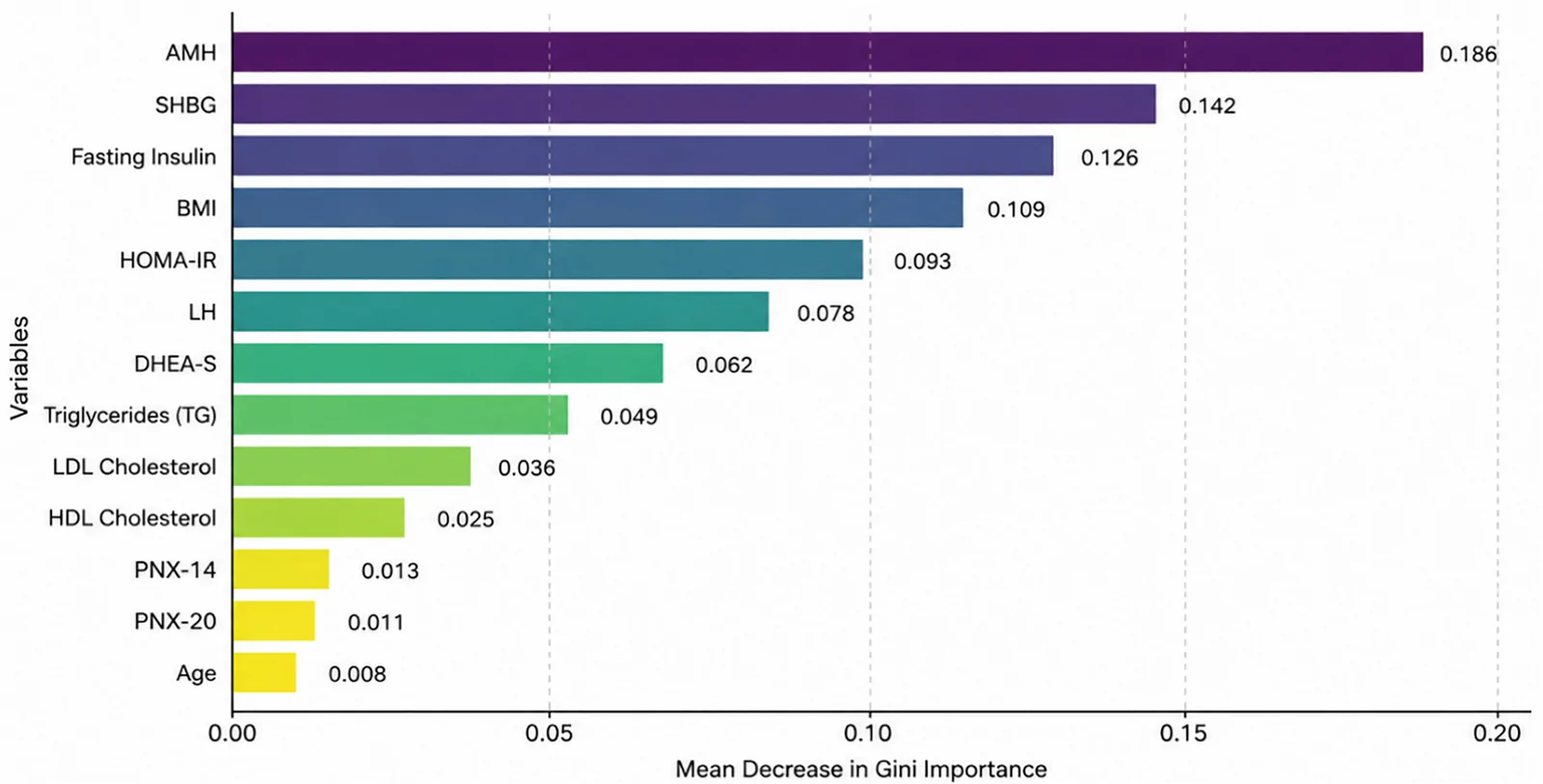
**Exploratory Random Forest feature-importance ranking of variables associated with PCOS.** The horizontal bar plot shows the relative importance of clinical, metabolic, and hormonal variables in an exploratory Random Forest analysis performed to identify variables associated with PCOS. Variable importance is expressed as feature-importance scores, with higher values indicating a greater relative contribution to model-based classification. AMH, SHBG, fasting insulin, and BMI demonstrated the highest importance scores, whereas PNX-14 and PNX-20 showed lower relative importance values (0.013 and 0.011, respectively), suggesting a modest contribution compared with established metabolic and hormonal markers. This analysis was conducted for exploratory feature-ranking purposes only and should not be interpreted as a validated predictive model or evidence of causal association. **Abbreviations:** AMH, anti-Müllerian hormone; BMI, body mass index; DHEA-S, dehydroepiandrosterone sulfate; HDL-C, high-density lipoprotein cholesterol; HOMA-IR, homeostatic model assessment for insulin resistance; LDL-C, low-density lipoprotein cholesterol; LH, luteinizing hormone; PCOS, polycystic ovary syndrome; PNX-14, phoenixin-14; PNX-20, phoenixin-20; SHBG, sex hormone–binding globulin.

## Discussion

The present study expands existing evidence regarding altered PNX levels in PCOS by simultaneously evaluating both biologically active isoforms, PNX-14 and PNX-20, alongside metabolic and reproductive parameters in a well-characterized cohort. Both isoforms exhibited marked elevation and significant associations with key metabolic and reproductive indicators, including AMH, insulin, and Homeostatic Model Assessment for Insulin Resistance (HOMA-IR). Importantly, PNX isoforms remained significantly associated with PCOS after multivariable adjustment, suggesting these associations are not solely explained by established metabolic and hormonal biomarkers. Nevertheless, given the close links between obesity, insulin resistance, and related metabolic disturbances with both PCOS and PNX levels, residual confounding cannot be entirely excluded. While mediation analysis hinted at potential interrelationships between PNX isoforms, AMH, and PCOS status, these findings warrant cautious interpretation and do not establish causal mediation or definitive biological mechanisms. The cross-sectional case-control design precludes causal inferences regarding a biological PNX-AMH axis. Therefore, these findings should be considered exploratory and hypothesis-generating rather than definitive evidence of a mechanistic pathway. The observed associations suggest that the relationship between PNX peptides and PCOS may involve complex interactions with hormonal and metabolic parameters, including AMH, BMI, and insulin resistance markers. Furthermore, as multiple exploratory analyses (correlation, clustering, Random Forest, and mediation analyses) were performed, the findings should be interpreted with caution. Longitudinal, mechanistic, and externally validated studies are required to clarify the biological significance and temporal relationships of these associations.

PCOS is increasingly recognized as a complex neuroendocrine-metabolic disorder, rather than solely a condition driven by ovarian dysfunction. Alterations in central and peripheral peptide signaling have been implicated in dysregulated gonadotropin-releasing hormone (GnRH) pulsatility and LH secretion, which may contribute to ovulatory dysfunction and hyperandrogenism [15–19]. These reproductive abnormalities are further influenced by insulin resistance and chronic low-grade inflammation, both of which contribute to the metabolic and endocrine disturbances characteristic of PCOS [20, 21].

PNX, a recently identified neuropeptide, has emerged as a potential modulator of reproductive and metabolic homeostasis. Experimental studies demonstrate that PNX may stimulate GnRH-mediated LH secretion and influence hypothalamic-pituitary-ovarian axis activity [8, 22]. Additionally, accumulating evidence suggests PNX may participate in glucose metabolism, insulin sensitivity, and inflammatory signaling pathways, indicating a broader systemic role [23–25]. Although PNX-14 has been reported to exhibit anti-inflammatory properties, its elevated levels in PCOS should be interpreted cautiously and may reflect a compensatory response to the chronic low-grade inflammation and metabolic dysregulation characteristic of the syndrome [26]. This apparent paradox may relate to context-dependent biological effects influenced by the underlying hormonal and metabolic environment. Thus, increased PNX-14 levels in PCOS may represent an adaptive association rather than definitive evidence of a protective biological mechanism.

Consistent with these observations, our findings demonstrated significantly elevated circulating PNX levels in women with PCOS. While PNX levels were positively associated with BMI and metabolic parameters, these associations require cautious interpretation, as the cross-sectional design precludes causal inference regarding PNX’s role in PCOS pathophysiology. Given substantial differences in BMI, insulin resistance, and related metabolic disturbances between PCOS and control groups, it remains challenging to fully disentangle whether elevated PNX levels are specifically related to PCOS or reflect broader metabolic dysfunction. The observed relationships may reflect broader neuroendocrine-metabolic alterations rather than a direct or dominant determinant of PCOS [27]. In this context, the relatively lower feature-importance scores observed in the Random Forest analysis further support a complementary rather than primary role for PNX in disease classification. Additionally, the positive association between PNX and LH may suggest a potential relationship with altered gonadotropin dynamics, consistent with previous reports describing PNX’s involvement in hypothalamic-pituitary-gonadal axis regulation [24, 28].

A particularly important finding of the present study was the observed association between PNX isoforms and AMH, a key regulator of folliculogenesis and a widely used biomarker in PCOS. While mediation analysis suggested an associative pattern involving AMH, these findings should be interpreted cautiously because mediation analysis within a cross-sectional case-control design cannot establish causal biological pathways. Potential AMH-independent mechanisms may include neuroendocrine effects on the hypothalamic-pituitary-gonadal axis, given previous evidence suggesting PNX may modulate GnRH-related LH secretion, as well as possible intraovarian effects related to follicular function [29, 30]. Additionally, observed associations with fasting insulin and HOMA-IR may indicate PNX’s relation to broader metabolic alterations in PCOS. However, the possibility that these associations represent epiphenomena rather than direct causal mechanisms cannot be excluded. While these hypotheses provide a biologically plausible interpretative framework for the observed associations [31], the temporal and causal relationships between PNX isoforms and PCOS cannot be established in the present study.

Clustering analysis further identified an exploratory data-driven cluster characterized by elevated PNX levels alongside increased AMH, insulin resistance, higher BMI, and adverse lipid profiles, accompanied by lower SHBG and HDL cholesterol levels. As the clustering variables were also used in cluster interpretation, these findings should be considered exploratory and interpreted cautiously. Nevertheless, this profile was enriched for women with PCOS and exhibited a more adverse metabolic and hormonal profile, suggesting PNX may be associated with broader neuroendocrine-metabolic dysregulation. However, the clinical significance of these exploratory clusters requires validation in independent cohorts.

From a metabolic perspective, the observed correlations between PNX isoforms and insulin resistance indices, triglycerides, and HDL cholesterol are consistent with previous evidence suggesting PNX’s potential involvement in glucose metabolism and inflammatory pathways [23–25]. These findings further support the concept of PCOS as a systemic neuroendocrine-metabolic disorder. However, Random Forest analysis demonstrated that established biomarkers, including AMH, SHBG, fasting insulin, and BMI, remained the most influential variables associated with PCOS, whereas PNX isoforms showed relatively lower feature-importance scores. Accordingly, these findings suggest PNX may play a complementary rather than primary role within the broader metabolic and hormonal profile of PCOS. Although ROC analyses demonstrated moderate discriminatory ability for PNX-14 and PNX-20, their relatively limited sensitivity and lower performance compared with established biomarkers such as AMH reduce their standalone clinical utility. Furthermore, because cutoff values and performance estimates were derived and evaluated within the same study population without internal or external validation, the possibility of overfitting and limited generalizability cannot be excluded. Therefore, these findings should be interpreted cautiously and considered exploratory until validated in independent cohorts.

PCOS is a highly heterogeneous condition characterized by distinct reproductive and metabolic phenotypes. However, formal stratification according to established PCOS phenotypes, including Rotterdam subtypes, was not performed in the present study, which may limit the interpretability and generalizability of the findings [14]. Additionally, the study cohort may have been enriched for individuals with metabolic and hyperandrogenic features, potentially limiting applicability across the broader spectrum of PCOS phenotypes [32]. Recent data-driven approaches have further emphasized the existence of biologically distinct PCOS subgroups with differing clinical and metabolic characteristics [33]. Therefore, the potential role of PNX isoforms as complementary biomarkers should be further evaluated across diverse and well-characterized PCOS phenotypes in larger independent cohorts.

Given the multiple exploratory analyses performed, including correlation, clustering, Random Forest, and mediation analyses, the findings should be interpreted cautiously and considered hypothesis-generating rather than definitive evidence of biological mechanisms.

### Strengths

This study has several notable strengths. A major strength is the simultaneous evaluation of both biologically active phoenixin isoforms together with comprehensive metabolic and hormonal profiling in women with PCOS. Additionally, the integration of advanced analytical approaches, including mediation analysis, PCA-based clustering, and Random Forest analysis, provided a multidimensional assessment of potential associations between PNX isoforms and PCOS-related features. While these analytical approaches offered complementary insights, they were primarily exploratory and do not overcome the inherent limitations of the observational cross-sectional study design. Nevertheless, the overall consistency of findings across multiple analytical approaches supports the internal coherence and robustness of the observed associations.

### Limitations

Several limitations should be acknowledged. The cross-sectional case-control design precludes causal inference and does not permit determination of temporal relationships between phoenixin isoforms, AMH, and PCOS. Accordingly, the observed associations and mediation findings should be interpreted as exploratory rather than evidence of definitive biological mechanisms. Although the sample size was adequate for the primary analyses, formal stratification according to established PCOS phenotypes was not performed, which may limit interpretation across different clinical subtypes. Residual confounding and selection bias cannot be entirely excluded despite adjustment for key metabolic variables, including BMI, insulin resistance, and lipid parameters. Furthermore, because elevated PNX levels may be related to broader metabolic dysfunction, it remains difficult to fully disentangle PCOS-specific effects from obesity- and insulin resistance-related influences.

Advanced statistical approaches, including mediation, clustering, and Random Forest analyses, were conducted for exploratory and hypothesis-generating purposes. Additionally, multiple exploratory analyses were performed without formal adjustment for multiple comparisons. Because internal validation procedures, multiplicity adjustment, bootstrap validation, train/test splitting, and cross-validation were not performed, the possibility of overfitting and type I error cannot be excluded. Similarly, ROC-derived cutoff values and diagnostic performance estimates were obtained and evaluated within the same cohort and therefore should be interpreted cautiously until externally validated. Potential measurement bias related to ELISA-based assays, including assay specificity and biological variability, should also be considered. Finally, the single-center design and relatively homogeneous study population may limit generalizability. Further longitudinal, mechanistic, and externally validated studies in larger multi-center cohorts are warranted.

### Future directions

Future studies should employ longitudinal and mechanistic designs to clarify the temporal and biological relationships between PNX isoforms and PCOS-related metabolic and reproductive alterations. Further investigation of potential interactions between PNX signaling, AMH regulation, and ovarian function is warranted. Larger, multi-center, and externally validated cohorts will also be necessary to determine the potential complementary value of PNX isoforms in characterizing biological and clinical heterogeneity within PCOS and multi-marker assessment approaches. Additionally, experimental and interventional studies targeting PNX-related pathways may provide further insight into their possible role in PCOS pathophysiology.

## Conclusion

Circulating PNX-14 and PNX-20 levels were significantly elevated in women with PCOS and were associated with key metabolic and reproductive parameters. These findings provide preliminary evidence supporting a potential relationship between phoenixin isoforms and PCOS-related reproductive and metabolic features. Although mediation analysis suggested associations between PNX isoforms, AMH, and PCOS status, the present findings should be interpreted as exploratory and associative rather than evidence of a causal mechanistic PNX-AMH axis. While PNX isoforms did not outperform established biomarkers such as AMH, their persistent association with PCOS after multivariable adjustment and their association with phenotypic clustering suggest a potential complementary role within multi-marker frameworks. Therefore, the incremental clinical value of PNX appears limited when used in isolation. Additionally, despite adjustment for key metabolic variables, including BMI and insulin resistance, the possibility of residual confounding cannot be excluded. Accordingly, the biological and clinical significance of PNX in PCOS remains to be established. Further longitudinal, mechanistic, and externally validated studies in larger independent cohorts are required to clarify the biological and clinical significance of these associations.

## Data Availability

The data that support the findings of this study are available on request from the corresponding author. The data is not publicly available due to privacy or ethical restrictions.
